# Response to the Letter to the Editor by Amee Patel

**DOI:** 10.48101/ujms.v126.8435

**Published:** 2021-12-31

**Authors:** Curt Ekström

**Affiliations:** Department of Neuroscience, Ophthalmology, Uppsala University, Uppsala, Sweden. http://orcid.org/0000-0002-8265-6518

Dear Editor,

We have noticed with great pleasure that our article has drawn attention among readers in the United Kingdom. The glaucoma survey in the rural district of Tierp, including 760 subjects 65–74 years of age, was a small study compared with many other population surveys. According to the cross-sectional character of the study, it was not possible to assess the effect of previous exposures at baseline. The presence of cataracts was ascertained based on retroillumination with lens opacities evident on slit-lamp examination. A detailed grading of the number of opacities in six stages was also performed. It is unlikely that any type of bias was involved in this part of the study. Nonetheless, as stated in the letter to *UJMS*, the lack of a standardised lens opacity grading system, like LOCS III, is a likely explanation to the moderate prevalence of cataracts found in the Tierp study. The limited sample size may also account for the lack of association with treated systemic hypertension. We have not discussed ethnicity in the article. However, the importance of exposure to sunlight was mentioned twice. We also discussed the association of myopia with lens opacities. To the best of my knowledge, only one population-based study has previously reported on cataract prevalence in Sweden, namely the Skövde Cataract Study, with the Tierp study being the second [1].

Curt Ekström, MD, PhD

Department of Neuroscience,

Ophthalmology, Uppsala University,

Uppsala, Sweden

http://orcid.org/0000-0002-8265-6518
